# Retinal OFF-Pathway Overstimulation Leads to Greater Accommodation-Induced Choroidal Thinning

**DOI:** 10.1167/iovs.62.13.5

**Published:** 2021-10-12

**Authors:** Hosein Hoseini-Yazdi, Scott A. Read, David Alonso-Caneiro, Michael J. Collins

**Affiliations:** 1 Contact Lens and Visual Optics Laboratory, Centre for Vision and Eye Research, School of Optometry and Vision Science, Queensland University of Technology, Brisbane, Queensland, Australia

**Keywords:** choroid, myopia, ON-/OFF-pathways, optical coherence tomography, refractive error

## Abstract

**Purpose:**

To examine the interactions between accommodation and overstimulation of the retinal ON- and OFF-pathways, and their association with changes in choroidal thickness (ChT) and vascularity.

**Methods:**

Optical coherence tomography imaging of the choroid of twenty young adults (ages 25 ± 5 years) was performed before and after a series of 30-minute-long viewing tasks, including reading a bright text on dark background (ON-pathway overstimulation) and dark text on bright background (OFF-pathway overstimulation), and a control task of viewing a movie with unbiased ON-/OFF-pathway activation. The viewing tasks were performed with relaxed, and 5 diopter (D) accommodation (induced by soft contact lenses) demands. Both reading texts were matched for the mean luminance (35 cd/m^2^), luminance contrast (87%), and letter size (approximately 11.8 arc minutes). The change in ChT from baseline associated with contrast polarity and accommodation was examined using linear mixed model analysis.

**Results:**

The subfoveal ChT decreased significantly by −7 ± 1 µm with 5 D accommodation compared with relaxed accommodation (−3 ± 1 µm; *P* < 0.001), and by −9 ± 1 µm with OFF-pathway compared with ON-pathway overstimulation (−4 ± 1 µm; *P* = 0.002) and the control condition (−2 ± 1 µm; *P* < 0.001). Overstimulation of the OFF-pathway, but not the ON-pathway, resulted in a significantly greater choroidal thinning compared with the control condition, both at relaxed (−7 ± 1 µm; *P* = 0.003) and 5 D (−11 ± 1 µm; *P* = 0.005) accommodation levels. Similar changes were also observed for macular total, stromal, and luminal ChT.

**Conclusions:**

Retinal OFF-pathway stimulation enhanced the choroidal thinning associated with accommodation, thereby providing a potential mechanism that involves accommodation and the retinal OFF-signaling pathway, linking near work and myopia.

The prevalence of myopia is increasing globally,^1^ with significant associations between myopia and more years spent in education consistently reported,^2^^–^^4^ and some studies suggesting a potential causal role for education in myopia development.^5^ The link between education and myopia may be explained by aspects of the intensive near work demands associated with education including, but not limited to, ocular accommodation.^6^ Short-term accommodation results in a transient increase in axial length (AxL) in both adults^7^^–^^16^ and children,^17^ and its cumulative effects could provide a basis for near activities resulting in longer term excessive axial elongation and the development of myopia.^18^^,^^19^

The choroid is involved in the vision-dependent processes regulating the growth and refractive state of the eye, with a thickening of the choroid associated with slowing of eye growth and development of hyperopia and a thinning of the choroid with increased eye growth and development of myopia.^18^^,^^20^ Although mechanical stretching of the globe associated with ciliary muscle contraction is thought to play a major role in ocular elongation during accommodation,^7^ there is also evidence suggesting a thinning of the choroid with accommodation,^9^^,^^13^^,^^16^ which accounts for about 35% of the accommodation-induced axial elongation of the eye.^13^^,^^16^

The visually guided mechanisms mediating changes in ChT are thought to be initiated locally by the retina.^21^^–^^24^ One such retinal mechanism is the relative activation of the ON- and OFF-pathways that exists at the synaptic level between the photoreceptors and bipolar cells in response to spatial and temporal light modulations.^25^^–^^31^ The ON-pathway responds to increments in light (e.g., rapid light increments such as bright letters on a dark background), whereas the OFF-pathway responds to decrements in light (e.g., rapid light decrements such as dark letters on a bright background).^32^ The human choroid responds to short-term overstimulation of the retinal ON- and OFF-pathways, when viewing distant visual targets, by demonstrating a thickening and thinning, respectively.^33^^,^^34^

It is suggested that spending more time on close distance reading, but not using computers, is associated with a greater odds ratio of myopia prevalence.^35^ Given that reading materials typically involve a printed or screen-based text with dark letters on a bright background (a visual stimulus that preferentially stimulates the OFF-pathway) and that the contrast polarity is typically varied randomly with other screen-based near tasks (e.g., computer games, browsing on computer screens at near), it might be that the greater levels of exposure to OFF-pathway stimulating materials during reading may underlie, at least partly, the greater risks associated with reading, compared with other near work tasks, on myopia.^33^^,^^34^ However, there is currently a lack of understanding of the role of OFF-pathway stimulation during accommodative reading tasks in the visually guided mechanisms regulating eye growth. This study therefore aims to examine the interactions between accommodation and retinal ON- and OFF-pathway overstimulation, and their association with changes in ChT, a well-established biomarker of myopia, and the vascularity of the choroid.

## Methods

### Participants

Twenty young adults with a mean age of 25 ± 5 years, including 10 emmetropes and 10 myopes, participated in this study. They were in good general health, had normal best corrected vision (logMAR 0.00 or better), normal binocular vision, monocular accommodation amplitude of more than 7 diopters (D), and no history or evidence of any ocular disease or surgery. Smokers and participants using medications and those using treatments to control the progression of myopia were excluded. No participants with astigmatic refractive errors of more than 1 D in either eye, or anisometropia of more than 1 D were included in the study to limit any potentially confounding effects from uncorrected astigmatic defocus,^36^ or anisometropia,^37^ upon the findings. The Queensland University of Technology human research ethics committee approved the study and all participants provided written informed consent and were treated in accordance with the tenets of the Declaration of Helsinki.

### Study Protocol

**Figure 1.**
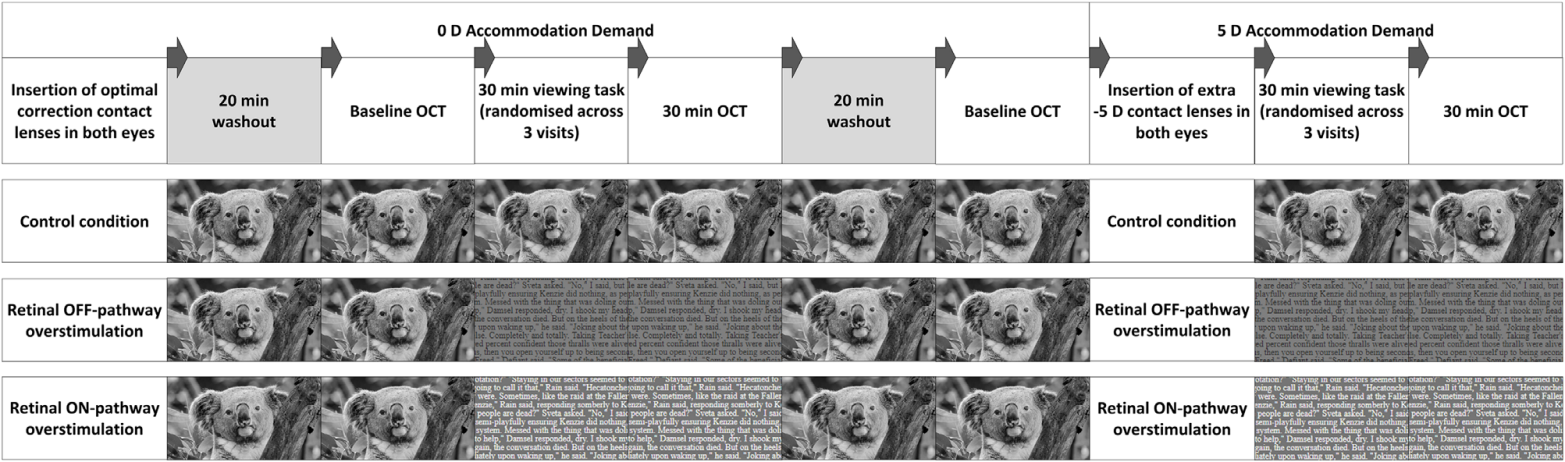
Overview of the study protocol to examine the ChT before and after a 30-minute viewing task at 0 D and 5 D accommodation demand using optical coherence tomography. The viewing tasks included a greyscale movie as the control condition, a text with dark letters on a bright background to overstimulate the retinal OFF-pathway or with bright letters on a dark background to overstimulate the retinal ON-pathway. A grey background was used instead of a white background for the OFF-pathway overstimulating text, and instead of a black background for the ON-pathway overstimulating text to match the mean luminance and the luminance contrast between the two contrast polarity conditions. The control condition, ON condition and OFF condition were tested over three separate visits in randomized order (i.e., total of six testing conditions).

Eligible participants attended three study visits on separate days, scheduled between 10 am and 2 pm, to decrease the influence of diurnal variations upon the findings.^38^^,^^39^ Each study visit comprised two 30-minute-long binocular viewing tasks with 0 D and 5 D accommodation demands, respectively, while wearing soft disposable spherical contact lenses (Proclear 1 Day, CooperVision Inc., San Ramon, CA), either providing optimal refractive correction with relaxed accommodation or inducing 5 D accommodation, with measures of ChT captured before and after each task. During each accommodation demand, a different visual stimulus was presented and viewed in primary gaze at a 3 m distance, including either text with bright letters on a dark background for ON-pathway overstimulation or dark letters on a bright background for OFF-pathway overstimulation, or a greyscale movie (hence approximately matched with the color cues provided in the text stimuli on the RGB screen) with no strong bias toward ON-/OFF-pathways as the control stimulus (Fig. 1). Each viewing task was preceded by a 20-minute period of watching a greyscale movie with relaxed accommodation to washout the influence of previous visual tasks^13^ and physical activity^40^ on ChT (Fig. 1).

The visual stimuli were projected on a screen encompassing a 30° × 20° visual field at a 3 m distance (similar to the 24° × 14° screen size used in a previous study^33^). The participants read through the text at a comfortable speed and maintained sharp focus on the text and the greyscale movie during all conditions. Consistent with a recent study,^33^ the mean luminance of both ON- and OFF-stimulating texts was 35 cd/m^2^, with a luminance contrast of 87%. However, the luminance of the control movie varied randomly throughout the presentation and between different visits. The visual angle of a capital letter in the text was 11.8 arc minutes during the 0 D accommodation tasks, and 11.6 arc minutes during the 5 D accommodation tasks (i.e., approximately 1.5% minification associated with the extra negative power in the contact lens to induce accommodation) using the Times New Roman font. Room illuminance was maintained at approximately 10 lux during all visits.

**Figure 2.**
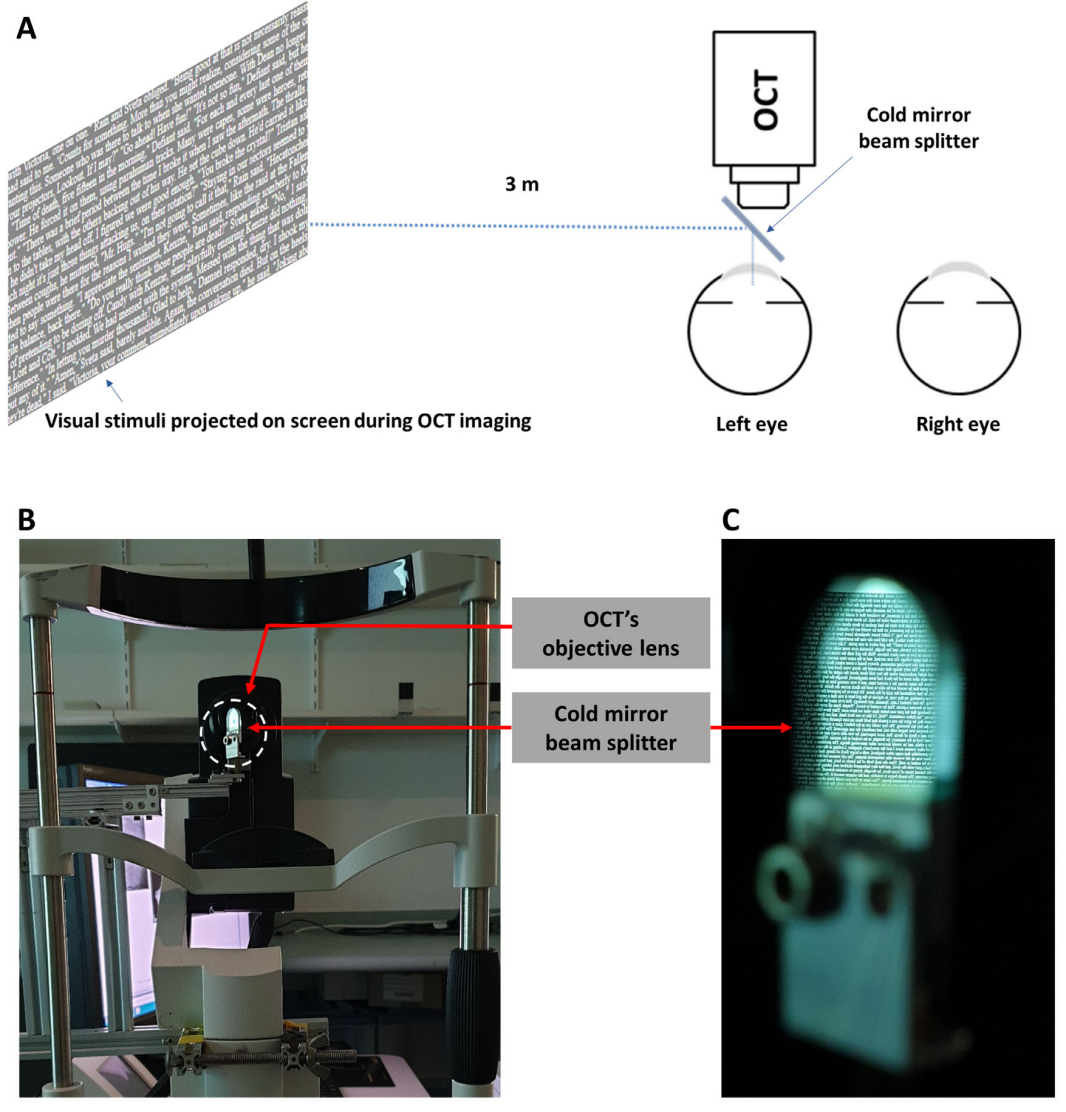
Schematic diagram (**A**) and front view image (**B**) illustrating the cold mirror beam splitter setup attached to the Spectralis OCT (and the Lenstar optical biometer) to allow simultaneous exposure to the three contrast polarity stimuli (presented at 3 m, ON-pathway stimulating text shown here) during the OCT imaging. The choroidal imaging was performed with the contact lens on-eye to induce 0 D or 5 D accommodation while a letter in the ON- or OFF-stimulating text, or the center of a Maltese cross in the control condition, was fixated (and maintained at sharp focus) through the beam splitter. The cold mirror beam splitter is surrounded by the white dashed circle in (**B**), and a magnified view of the screen as seen through the beam splitter is shown in (**C**). The appropriate level of accommodation was verified objectively by the examiner through obtaining a focused en face retinal image, with minimal refocusing of the instrument required to compensate the power of the extra negative contact lens during the viewing tasks with accommodation compared with those with relaxed accommodation.

High-resolution enhanced-depth imaging optical coherence tomography (OCT) of the left eye's posterior segment was performed across the horizontal macular region, centered on the fovea, using the Spectralis OCT device (Heidelberg Engineering Co., Jena, Germany) to provide measures of ChT before and after each viewing task. Three B-scans were acquired at each time point, each being an average of 100 frames,^41^ and captured with the follow-up function of the instrument. To allow simultaneous exposure to the three contrast polarity stimuli during the OCT imaging, a cold mirror beam splitter was mounted on the instrument, while the choroidal imaging was performed with the contact lens on-eye to induce 0 D or 5 D accommodation (Fig. 2). The Spectralis instrument's focus data of the retinal en face image indicated that a minimal refocusing of the instrument was required to compensate the power of the extra negative contact lens during the viewing tasks with accommodation (mean ± standard deviation of the focus of the retinal en face image, +0.24 ± 0.50 D) compared with those with relaxed accommodation (+0.05 ± 0.20 D), thus confirming that accommodation had taken place during the OCT image acquisition. During the OCT imaging, the blue internal fixation target of the instrument was switched off, and a letter in the ON- or OFF-stimulating text, or the center of a Maltese cross in the control condition, was fixated (and maintained in sharp focus) through the beam splitter (Fig. 2).^21^

Before and after each viewing task and immediately after the OCT imaging, the accommodative response of the left eye was confirmed by measuring the anterior chamber depth (ACD) and crystalline lens thickness (LT) using the Lenstar LS-900 (Haag Streit AG, Köniz, Switzerland) optical biometer equipped with a cold mirror beam splitter (similar to the Spectralis setup) (Fig. 2) to maintain exposure to the contrast polarity and accommodation stimuli. Five measurements were collected consecutively, ensuring that the external fixation target viewed through the beam splitter was sharply focused and aligned with the dim red internal fixation target. Given that the presence of the beam splitter during optical biometry decreased the signal strength from the anterior and posterior surfaces of the crystalline lens, the ACD and LT could be measured in 55% to 90% and 50% to 80% of times across all measurement time points, respectively.

### Data Analysis

**Figure 3.**
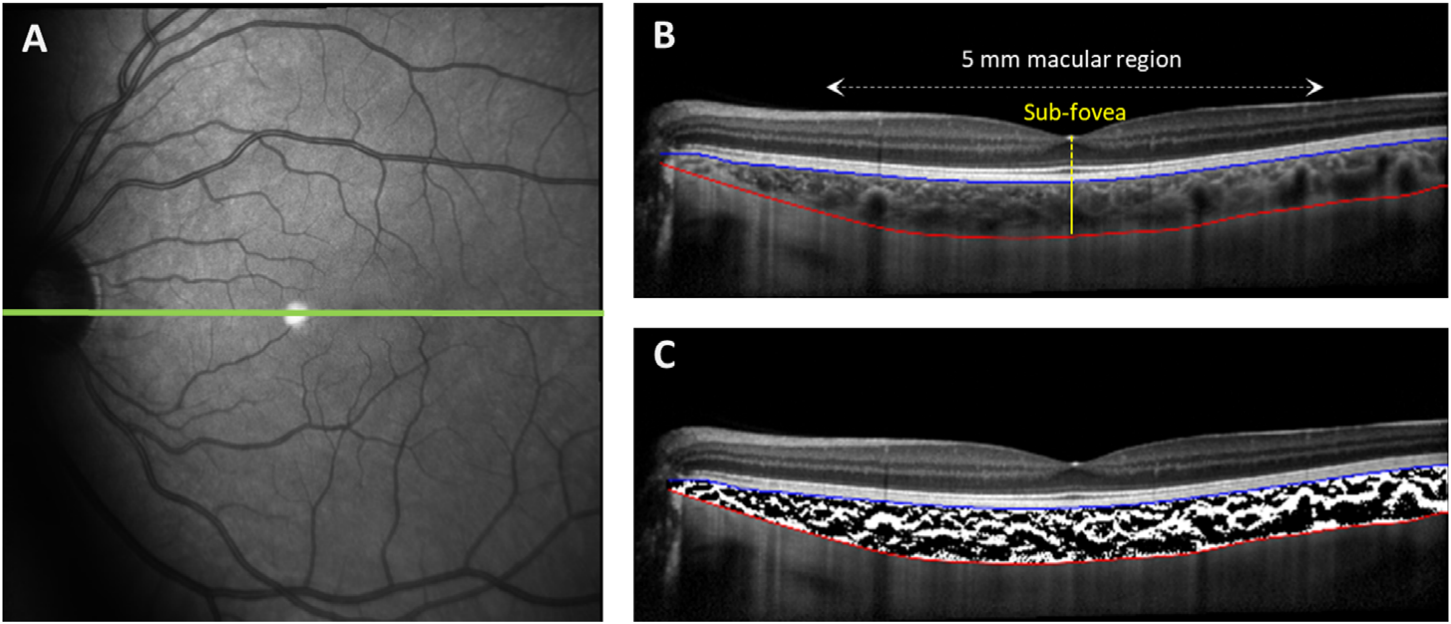
Illustration of the fundus en face image and the position of the 30° OCT scan (*green horizontal line* in **A**) and the corresponding high-resolution segmented enhanced depth imaging B-scan along the horizontal meridian (**B** and **C**) in a representative participant. The ChT was measured between the hyper-reflective line corresponding to the inner surface of the retinal pigment epithelium and Bruch's membrane complex (*blue line* in **B** and **C**) and the hyper-reflective line corresponding to the inner surface of the choroidoscleral interface (*red line* in **B** and **C**) at the subfovea (*solid yellow* line in **B**) below the deepest point in the foveal pit (*dashed yellow line* in **B**) and across the central 5-mm macular region (*dashed double* *arrow white line* in **B**). The binarized view of the choroid in the same segmented B-scan is shown in (**C**).

The three B-scans collected at each measurement time point were exported and segmented using a semiautomatic procedure^42^^,^^43^ by one experienced masked observer. The transverse magnification of the B-scans was then adjusted to account for variations in AxL (Supplementary Material). The ChT was measured subfoveally at the deepest point of the foveal pit and averaged across the central 5-mm macular region (Figs. 3A, B).

Using a custom-written program, the segmented B-scans were binarized using the Niblack local binarization method (Fig. 3C).^44^ In this method, the binarization of the OCT image is based on the local pixel brightness in smaller subregions in the image, known as the analysis window.^45^^,^^46^ Two masked observers determined the optimal window size independently for each B-scan, through manual adjustments of the analysis window size to provide an optimal visualization of the choroidal stromal and luminal regions in the binarized images. The results from the two observers were in good agreement (intraclass correlation coefficient, 0.942; 95% confidence interval, 0.926–0.955; *P* < 0.001; mean ± standard error of mean, observer 1, 41 ± 4 pixels, observer 2, 42 ± 4 pixels; *P* = 0.17) and were averaged for each individual participant at each measurement time point and used for calculation of the choroidal vascularity index (CVI). The CVI was calculated as the ratio of the dark pixels (signifying luminal regions) to the total number of pixels,^47^ and averaged across the 5-mm macular region. The mean macular CVI for each B-scan was then used to calculate the macular luminal (CVI × ChT) and stromal thickness (total ChT – luminal thickness) of the choroid.

Using IBM SPSS Statistics 26 (www.ibm.com/software/analytics/spss), separate linear mixed model (LMM) analyses were conducted to examine the changes in ACD, LT, subfoveal ChT, and choroidal parameters averaged across the macula associated with contrast polarity, accommodation level, and refractive error group. One female emmetropic participant did not show evidence of active accommodation (ACD and LT changes of <150 µm) and the data obtained during accommodation were not included in the LMM analyses. All possible factorial interactions were included as fixed factors and the slope and intercept of individual subjects were included as random factors in each LMM. A compound symmetry covariance structure was assumed for the repeated factors (contrast polarity and accommodation) and a variance components covariance structure was assumed for the random factors. Bonferroni corrected post hoc tests were conducted for significant main effects and interactions. Since the study protocol involved the 0 D accommodation condition with exposure to ON- or OFF-stimulating text, followed by a 20-minute washout period, additional LMM analyses were conducted separately for each choroidal parameter to examine the recovery of changes associated with contrast polarity following 20 minutes after the completion of the tasks with relaxed accommodation. Pearson correlation analysis was also conducted separately for each polarity and accommodation condition to examine the association between the baseline thickness of the choroid (subfoveal and macular) and the magnitude of change in ChT. The change from baseline for all ocular parameters are represented as the mean ± standard error of the mean.

## Results

The demographics and the baseline ocular characteristics of the participants are displayed in Table 1.

**Table 1.** Demographics and Ocular Characteristics of the Studied Participants

	All (*n* = 20)	Emmetropes (*n* = 10)	Myopes (*n* = 10)	*P* Value: Emmetropes vs. Myopes
Age (years)	25 ± 5	24 ± 4	25 ± 6	0.46^*^
	[20, 35]	[20, 31]	[21, 35]	
Spherical equivalent	−1.5 ± 2.2	+0.12 ± 0.33	−3.12 ± 2.03	**0.001** ^*^
Refractive error (D)	[−6.00, 0.75]	[−0.25, 0.75]	[−6.00, −1.00]	
Sex (%, F:M)	60:40	50:50	70:30	0.32^†^
Subfoveal ChT (µm)	347 ± 93	414 ± 60	280 ± 68	**<0.001** ^*^
	[197, 525]	[324, 525]	[197, 428]	
Macular ChT (µm)	322 ± 81	377 ± 59	267 ± 62	**0.001** ^*^
	[184, 506]	[298, 506]	[184, 403]	
Macular luminal thickness (µm)	185 ± 42	213 ± 32	158 ± 32	**0.001** ^*^
	[115, 282]	[178, 282]	[115, 227]	
Macular stromal thickness (µm)	137 ± 42	164 ± 31	109 ± 33	**0.001** ^*^
	[64, 224]	[119, 224]	[64, 176]	
Macular CVI (%)	58 ± 5	57 ± 3	60 ± 6	0.15^*^
	[50, 70]	[50, 60]	[52, 70]	
ACD (mm)	3.25 ± 0.26	3.21 ± 0.16	3.29 ± 0.34	0.52^*^
	[2.97, 4.09]	[2.97, 3.53]	[2.98, 4.09]	
LT (mm)	3.51 ± 0.18	3.53 ± 0.17	3.50 ± 0.20	0.75^*^
	[3.21, 3.93]	[3.32, 3.93]	[3.21, 3.74]	

All parameters, except sex, are presented as mean ± standard deviation with minimum and maximum values in brackets. The choroidal parameters are derived from the average of the baseline values across all conditions. The macular choroidal parameters were measured across the central 5-mm region centered on the fovea. The ACD and LT are derived from the average of the baseline values available in 60%–85% and 50%–75% of times across all conditions.

* *P* value from an independent Student *t* test. Statistically significant *P* values are presented in bold.

† *P* value from a χ^2^ test.

The ACD and LT underwent statistically significant changes associated with accommodation (both *P* < 0.001), with these accommodation-induced changes not differing significantly between contrast polarities (both *P* > 0.05). The ACD decreased by −327 ± 26 µm and the LT increased by 296 ± 28 µm with a 5 D accommodation demand, consistent with the typical changes reported for these parameters with accommodation in young adults.^13^^,^^14^

### Subfoveal and Macular ChT

Significant changes in the subfoveal ChT were observed associated with contrast polarity (F_2, 88_ = 14.63; *P* < 0.001) and accommodation (F_1, 90_ = 21.21; *P* < 0.001). Averaged across both accommodation levels and both refractive error groups, the subfoveal ChT decreased by −9 ± 1 µm with OFF-pathway compared with ON-pathway overstimulation (−4 ± 1 µm; *P* = 0.002) and the control condition (−2 ± 1 µm; *P* < 0.001). Averaged across all contrast polarities and refractive error groups, the subfoveal ChT decreased by −7 ± 1 µm with a 5 D accommodation compared with relaxed accommodation (−3 ± 1 µm; *P* < 0.001).

**Figure 4.**
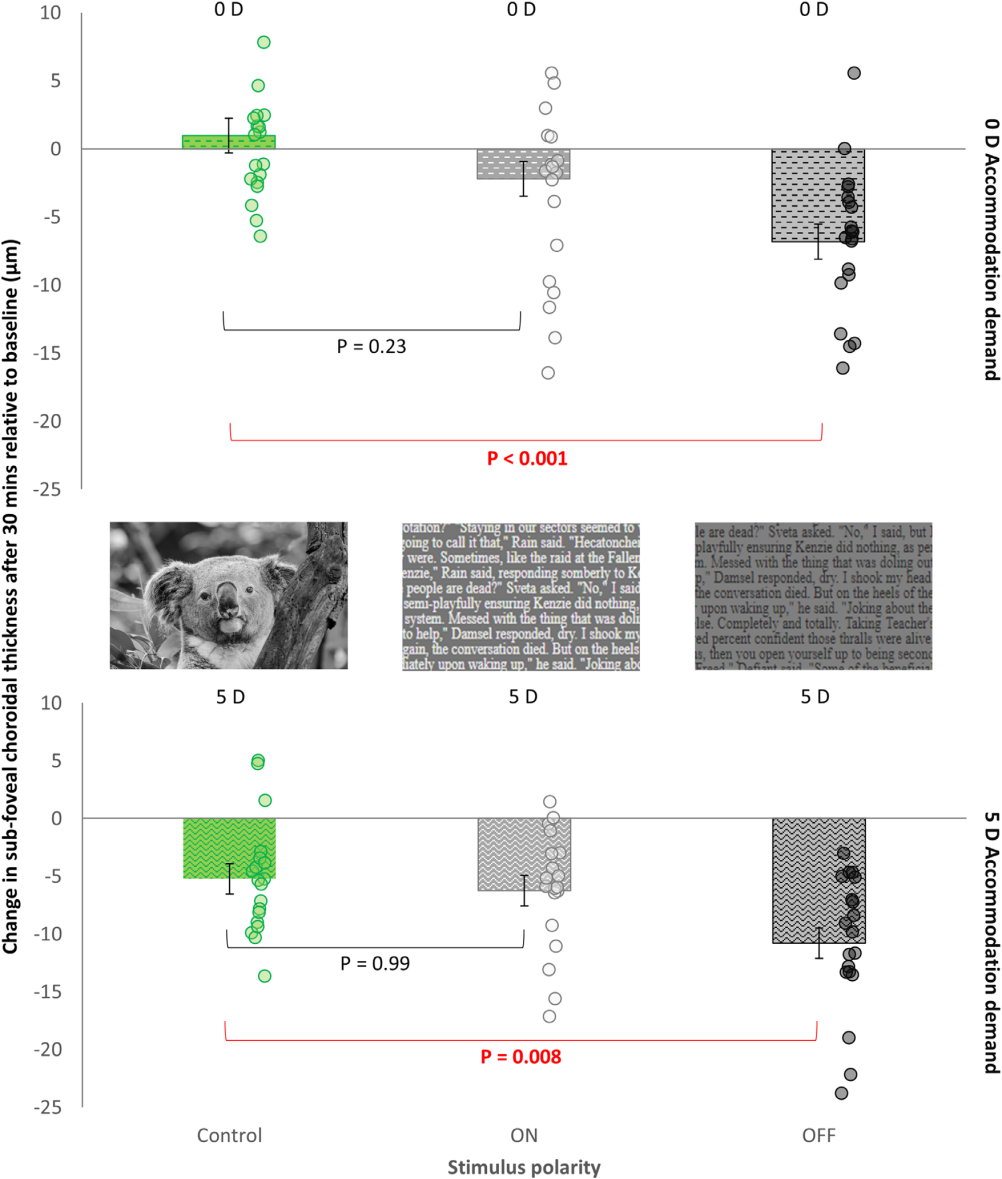
The change in the subfoveal ChT after 30 minutes of exposure to different contrast polarities at 0 D (*top*) and 5 D (*bottom*) accommodation demands. Positive and negative values on the *y*-axis indicate a thickening and a thinning of the choroid, respectively. The error bars indicate the standard error of the mean. Individual changes in the subfoveal ChT are represented by the circle symbols in each graph.

The contrast polarity by accommodation interaction (F_2, 88_ = 0.50; *P* = 0.61) and the contrast polarity by accommodation by refractive error group interaction (F_2, 88_ = 1.49; *P* = 0.23) were not significant, suggesting that the subfoveal ChT changes in response to various contrast polarities were similar across both accommodation demands and refractive error groups. Averaged across both refractive error groups, overstimulation of the OFF-pathway, but not the ON-pathway, resulted in a greater decrease in subfoveal ChT compared with the control condition, both at 0 D (−7 ± 1 µm; *P* < 0.001) and 5 D (−11 ± 1 µm; *P* = 0.008) accommodation demands (Fig. 4, Table 2).

**Table 2.** The Group Mean Change (Standard Error of the Mean) In Measures of ChT Parameters (µm) and Vascularity Index (%) in Response to a 30-Minute Viewing of Different Contrast Polarities at 0 D and 5 D Accommodation Demands

		Contrast Polarity			*P* Value^*^			
Change in Ocular Parameters	Accommodation Demand	CTRL	ON	OFF	Accommodation^†^	Contrast Polarity^†^	Control vs. ON	Control vs. OFF
Subfoveal ChT (µm)	0 D	1 (1)	−2 (1)	−7 (1)	**<0.001**	**<0.001**	0.23	**<0.001**
	5 D	−5 (1)	−6 (1)	−11 (1)			0.99	**0.008**
	***P* value** ^*^ **5 D vs. 0 D**	**0.001**	**0.03**	**0.03**				
Macular ChT (µm)	0 D	1 (1)	−2 (1)	−7 (1)	**<0.001**	**<0.001**	0.13	**<0.001**
	5 D	−6 (1)	−7 (1)	−11 (1)			0.99	**0.005**
	***P* value** ^*^ **5 D vs. 0 D**	**0.001**	**0.002**	**0.007**				
Macular luminal thickness (µm)	0 D	0 (1)	−2 (1)	−3 (1)	**<0.001**	**0.008**	0.12	**0.01**
	5 D	−5 (1)	−5 (1)	−6 (1)			0.99	0.39
	***P* value** ^*^ **5 D vs. 0 D**	**<0.001**	**0.015**	0.06				
Macular stromal thickness (µm)	0 D	0 (1)	0 (1)	−3 (1)	**0.003**	**<0.001**	0.99	**0.001**
	5 D	−1 (1)	−3 (1)	−4 (1)			0.64	**0.01**
	***P* value** ^*^ **5 D vs. 0 D**	0.09	**0.02**	0.26				
CVI (%)	0 D	−0.1 (0.2)	−0.2 (0.2)	0.2 (0.2)	0.31	0.20	–	–
	5 D	−0.3 (0.2)	−0.1 (0.2)	−0.1 (0.2)			–	–
	***P* value** ^*^ **5 D vs. 0 D**	–	–	–				

* *P* values are adjusted for multiple comparisons using Bonferroni method.

† Main effects of accommodation and contrast polarity from a LMM analysis. Statistically significant *P* values are presented in bold. Pairwise comparisons were only performed for metrics exhibiting significant main effects in the LMM analysis. The macular choroidal parameters were measured across the central 5-mm region centered on the fovea.

Similarly, statistically significant changes in measures of macular ChT were found associated with contrast polarity and accommodation (both *P* < 0.001) (Table 2), but the contrast polarity by accommodation and the contrast polarity by accommodation by refractive error group interactions were again not statistically significant (both *P* > 0.05). The observed changes in the subfoveal ChT and macular ChT were not significantly associated with the baseline thickness of the choroid in any polarity or accommodation condition (all *P* > 0.05).

### Choroidal Vascularity Parameters

Measures of CVI were not altered significantly in response to changes in contrast polarity (F_2, 88_ = 1.65; *P* = 0.20) or accommodation (F_1, 89_ = 1.06; *P* = 0.31), suggesting that quantitatively similar changes occurred in the luminal and stromal components associated with these two experimental conditions. Further analyses did reveal statistically significant main effects of contrast polarity and accommodation for the changes in both luminal and stromal thicknesses (all *P* < 0.01, Table 2), although the contrast polarity by accommodation and the contrast polarity by accommodation by refractive error group interactions were not significant (all *P* > 0.05). Post hoc comparisons between the two accommodation demands revealed a significant decrease in luminal (−5 ± 1 µm; *P* < 0.001), but not stromal thickness (−1 ± 1 µm; *P* = 0.09), with accommodation during unbiased stimulation of the ON-/OFF-pathways (Table 2).

### Recovery of ON-/OFF-Pathway–Induced Choroidal Changes During Relaxed Accommodation

**Figure 5.**
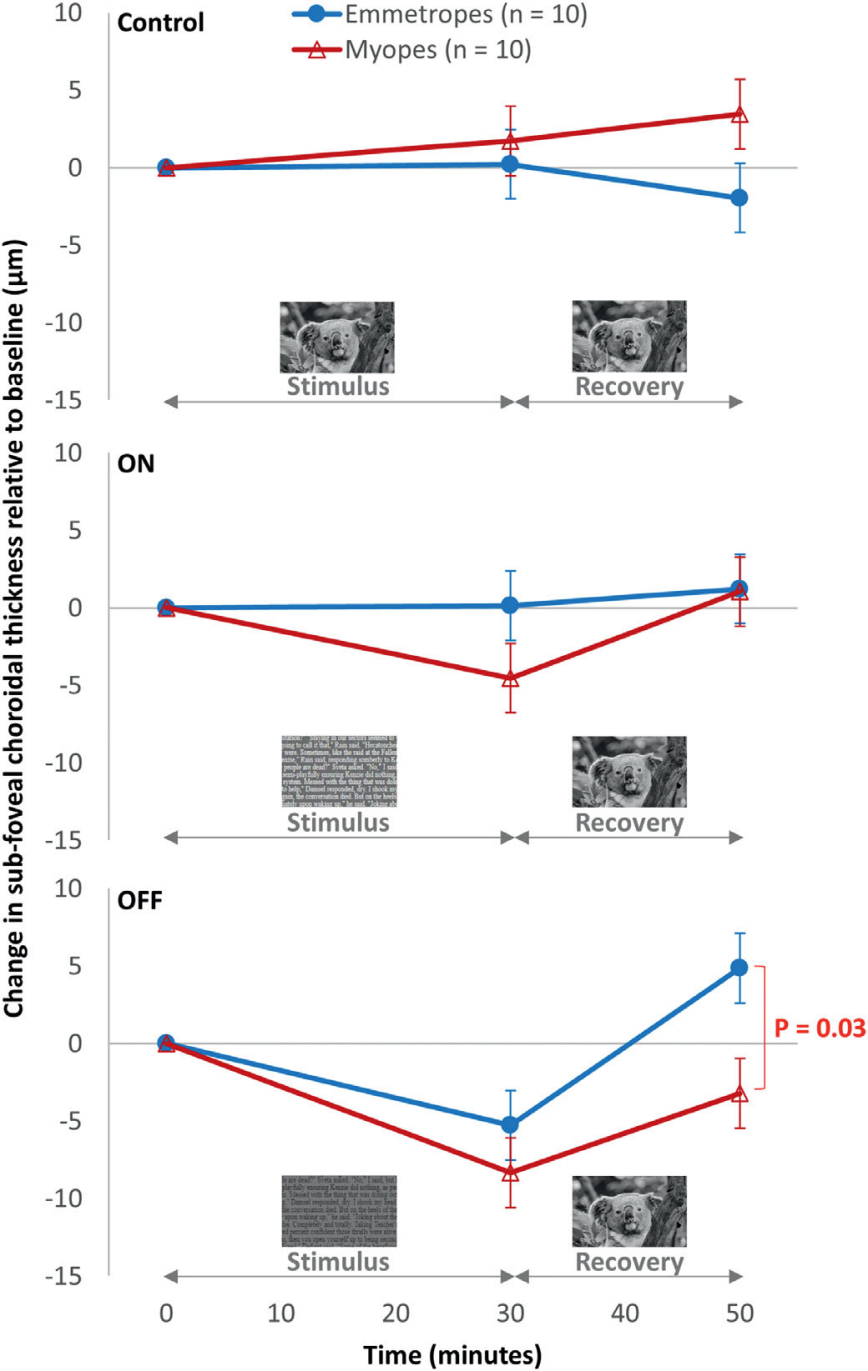
Comparison of changes in the subfoveal ChT after 30 minutes of exposure to control (*top*), ON (*middle*), and OFF (*bottom*) contrast polarities at 0 D accommodation demand and after 20 minutes of recovery between emmetropes (*blue closed circles*; *n* = 10) and myopes (*red open triangles*; *n* = 10). The error bars indicate the standard error of the mean.

Subfoveal ChT and all other choroidal parameters recovered to the baseline thickness 20 minutes after exposure to different contrast polarities was ceased (main effect of polarity; all *P* > 0.98; change relative to baseline <2 µm for all thickness parameters and <0.5% for CVI across all polarities). However, a statistically significant contrast polarity by refractive error group interaction was found for recovery of contrast polarity-induced changes in subfoveal ChT (F_2, 36_ = 4.82; *P* = 0.01) and macular ChT (F_2, 36_ = 3.46; *P* = 0.04). The decrease in ChT associated with OFF-pathway stimulation was sustained in myopes (change relative to baseline: subfovea, −3 ± 3 µm; macula, −2 ± 2 µm) but not in emmetropes (subfovea, 5 ± 3 µm; macula, 3 ± 2 µm) after the 20-minute washout (recovery) period, with the difference in ChT changes between myopes and emmetropes being statistically significant for subfoveal (*P* = 0.03) (Fig. 5C), but not macular measurements (*P* = 0.1).

## Discussion

This study provides novel insights into the interaction between changes in thickness and vascularity of the choroid in response to short-term overstimulation of the retinal ON- and OFF-pathways and ocular accommodation. We found that overstimulation of the retinal OFF-pathway, but not the ON-pathway, caused a significant decrease in ChT during relaxed accommodation and significantly enhanced the choroidal thinning associated with ocular accommodation. The magnitude of decrease in ChT with accommodation combined with OFF-pathway stimulation was approximately double that observed with accommodation alone.

### Choroid Response to ON-/OFF-Pathway Overstimulation During Relaxed Accommodation

In this study, short-term overstimulation of the retinal OFF-pathway through reading a standard polarity text for 30 minutes with relaxed accommodation caused a significant thinning of both the subfoveal and macular choroid compared with the control condition with unbiased stimulation of the ON-/OFF-pathways. The magnitude of choroidal thinning with OFF-pathway overstimulation was not significantly different between emmetropes and myopes, consistent with the findings from a recent study.^48^ The decrease in human ChT associated with short-term retinal OFF-pathway overstimulation was first reported by Aleman et al.^33^ in young adults after reading a standard polarity text for 60 minutes and was later confirmed by Wang et al.^34^ after short-term temporal stimulation of the retinal OFF-pathway.

This study further revealed that the choroidal thinning associated with retinal OFF-pathway stimulation results from a significant decrease in both the luminal and stromal components of the choroid. Given the evidence that the OFF-pathway induced thinning of the choroid in chicks was associated with relative decrease in levels of retinal dopamine (compared with ON-pathway stimulation),^34^ it seems possible that this mechanism also affected both the stromal and vascular tissues of the choroid in young adults in this study. Collectively, these results are consistent with the hypothesis that preferential stimulation of the retinal OFF-pathway may be associated with dopaminergic mechanisms that contribute to myopic eye growth.

Preferential stimulation of the retinal ON-pathway slows down eye growth in chicks, resulting in relative hyperopia compared with preferential stimulation of the retinal OFF-pathway,^25^ whereas a deficit in the ON-pathway signaling promotes myopic eye growth in mice^28^^,^^30^ and humans.^49^ This study did not find a significant change in either the subfoveal or macular ChT after short-term overstimulation of the retinal ON-pathway, through reading a reversed polarity text (letter size approximately 11.8 arc minutes) for 30 minutes with relaxed accommodation, compared with the control condition with unbiased stimulation of the retinal ON-/OFF-pathways. In contrast, Aleman et al.^33^ found an increase in the subfoveal ChT (approximately 5 and 10 µm) with ON-pathway overstimulation after a 30- and 60-minute reading of a reversed polarity text (letter size approximately 11.8 arc minutes) with relaxed accommodation, using a similar OCT device and a similar mesopic illumination level to our study. The reason for the apparent discrepancy in these results is unclear. In the study by Aleman et al.,^33^ the eye was not exposed to the ON-pathway overstimulating text during OCT imaging, but was presumably exposed to the luminous blue fixation target of the OCT device. Previous studies suggest that brief stimulation (approximately 1 minute) of the melanopsin-driven retinal pathway with blue light upregulates the ON-pathway–mediated response from the dopaminergic retinal amacrine cells,^50^ and increases the vitreal dopamine levels.^51^ Therefore, we hypothesize that the exposure of the eye to the blue fixation light in the study by Aleman et al.^33^ might have altered the ON-pathway signaling or triggered other short wavelength–sensitive retinal signaling pathways,^50^^,^^52^ resulting in the observed thickening of the choroid.

Indeed, a recent report of a follow-up study did not show a significant change in the AxL with a 30-minute exposure to a similar (small sized) ON-pathway stimulating text in mesopic illumination using an optical biometer with a red fixation target.^48^ The ON-pathway stimulation is thought to exert its effects on ChT through dopaminergic amacrine cells,^34^ with animal studies also showing an increased dopamine release from these cells with exposure to light,^31^^,^^53^ and decreased dopamine release from these cells with ON-pathway stimulation in low light levels.^54^^,^^55^ It is plausible, therefore, that the ambient lighting conditions may interact with the ON-pathway stimulation and influence the choroidal changes observed.

Further, a crossover inhibition of the ON- and OFF-pathways through amacrine cell inputs has been observed, with ON-pathway inhibition occurring when OFF-pathway stimulation occurs and vice versa.^56^ Also, movements of the eye seem to provide temporal cues that contribute to the vision-dependent mechanisms regulating the eye growth.^57^ Therefore, the fixational eye movements during reading of small text in low light levels may have provided temporal cues to the retina resulting in overstimulation of the OFF-pathway with possible associated inhibitory effects on the ON-pathway signaling. This finding is consistent with evidence on the increased asymmetric signaling of the ON-/OFF-retinal pathways in mesopic conditions resulting in greater OFF-pathway responses^58^ and also the evidence suggesting that small, fast, dark stimuli overstimulate the OFF-pathway and understimulate the ON-pathway.^59^

Studies in humans^60^ and primates^61^ also show that the ON retinal ganglion cells have a larger receptive field than the OFF retinal ganglion cells, suggesting that the small text size used in our study may have understimulated the ON-pathway. Indeed, Schaeffel et al.^48^ have reported a significant decrease in AxL (consistent with an increase in ChT) in response to reading a reversed polarity ON-stimulating text with relaxed accommodation only when the text size was large, but not when it was small.^48^ Further research is needed to better understand the optimal conditions that stimulate the retinal ON-pathway for its possible antimyopiagenic effects in humans by examining the ChT and AxL changes associated with ON- and OFF-pathway signaling under various lighting conditions and different sized stimuli.

### Recovery of Choroid Response to ON-/OFF-Pathway Overstimulation During Relaxed Accommodation

Changes in choroidal parameters associated with overstimulation of the retinal ON-/OFF-pathways during relaxed accommodation were transient since most parameters had recovered to their baseline value within 20 minutes after exposure to ON-/OFF-stimulation was ceased. The only exception was observed for changes in subfoveal ChT, with the observed thinning associated with OFF-pathway stimulation being sustained in myopes, but not in emmetropes, after the 20-minute recovery period. The sustained OFF-pathway induced choroidal thinning in myopes is a novel finding in this study and may be associated with more potent OFF-pathway–mediated signals or less potent ON-pathway–mediated signals in myopes. The slower recovery of the accommodation-induced AxL elongation previously observed after a standard polarity text (OFF-stimulating) reading task in myopes than emmetropes^13^ may also be explained by this hypothesis.

### Choroid Response to Unbiased ON-/OFF-Pathway Stimulation During Accommodation

Short-term accommodation during unbiased stimulation of the retinal ON-/OFF-pathways caused a significant decrease in both the subfoveal and macular ChT compared with the control condition with relaxed accommodation. The accommodation-induced thinning of the choroid has also been reported by previous studies^13^^,^^16^ and the magnitude of choroidal change in our current study is consistent with these previous reports. A range of factors have been proposed to be involved in the choroidal thinning with accommodation, including the stretching of the globe owing to intraocular and extraocular mechanical forces^7^^,^^62^^,^^63^ and optical factors, including decreased retinal image quality^64^ and increasing levels of hyperopic defocus and negative spherical aberrations during accommodation.^65^

The current study provides further evidence regarding changes in the vascularity of the choroid associated with accommodation. Although the CVI did not change significantly with accommodation, a separate analysis of the luminal and stromal thicknesses revealed that the accommodation-induced choroidal thinning was attributed to a significant decrease in the luminal, but not the stromal, thickness of the choroid during unbiased stimulation of the ON-/OFF-pathways (Table 2). Therefore, thinning of the choroidal vasculature may have a greater role than stromal changes in modulating ChT with accommodation (at least in the horizontal meridian of the macular region), possibly through contractile forces imposed by the nonvascular smooth muscle cells of the choroid^16^^,^^66^ and/or modulation of the choroidal blood flow mediated by increased parasympathetic input from accommodation.^67^^,^^68^

### Choroid Response to ON-/OFF-Pathway Overstimulation During Accommodation

A novel finding in this study is that the accommodation-induced thinning of the choroid doubled when the retinal OFF-pathway was also preferentially stimulated during accommodation, through reading a standard polarity text, compared with when equal stimulation of the ON-/OFF-pathways occurred during accommodation. The accommodation-induced changes in the ChT or AxL with differential stimulation of the retinal ON-/OFF-pathways have not been explored systematically to date, because these responses were only examined with equal stimulation of the ON-/OFF-pathways, by using a Maltese cross or a movie as a visual target,^9^^,^^12^ or presumably with greater stimulation of the OFF-pathway, by using a black on white background text as a visual target^8^^,^^13^ during induced accommodation. Interestingly, the magnitude of increase in the AxL (approximately 8 µm)^12^^,^^17^ or decrease in the ChT (approximately 5 µm)^16^ associated with accommodation during unbiased stimulation of ON-/OFF-pathways (approximately 6 D demand) seems to be smaller than the magnitude of axial elongation (approximately 15–20 µm)^8^^,^^13^ or choroidal thinning (approximately 8 µm)^13^ during preferential stimulation of the OFF-pathway with accommodation (approximately 4–5 D demand). The magnitude of choroidal thinning is thought to provide a biomarker for longer term excessive myopic eye growth.^69^^,^^70^ Therefore, our results showing an increase in the accommodation-induced thinning of the choroid during OFF-pathway stimulation imply that the myopiagenic effects associated with near work are potentially enhanced with simultaneous overstimulation of the retinal OFF-pathway, such as during reading a text with black letters on a bright background in low light levels. Further research is required to explore the impact of ON- or OFF-pathway overstimulation during various near tasks on the incidence and prevalence of myopia to provide insights into the clinical significance of contrast polarity during near work.

## Conclusions

A significant thinning of the choroid was found in response to short-term stimulation of the retinal OFF-pathway during relaxed accommodation in the human eye. It was also found that retinal OFF-pathway stimulation enhanced the choroidal thinning associated with accommodation, thereby providing a potential mechanism that involves accommodation and the retinal OFF-signaling pathway, linking near work and myopia.

## Supplementary Material

Supplement 1
