# Supplementary material for: Retinal OFF-Pathway Overstimulation Leads to Greater Accommodation-Induced Choroidal Thinning
Source: Invest Ophthalmol Vis Sci. 2021 Oct 12;62(13):5. doi: 10.1167/iovs.62.13.5 (PMC8525845; doi:10.1167/iovs.62.13.5)
Supplement: Supplement 1 [file iovs-62-13-5_s001.pdf]

Procedure for adjusting the transverse magnification of the B-scans:

The anterior chamber depth and crystalline lens thickness could not be measured in 10 – 45 % and 20 – 50 % of times across all measurement time points, respectively, due to a reduced signal strength from the anterior or posterior surfaces of the crystalline lens associated with the presence of the beam splitter during optical biometry. Therefore, the transverse magnification of the B-scans collected during relaxed accommodation was adjusted using only the axial length of the eye, measured from the anterior surface of the contact lens to the retinal pigment epithelium with Lenstar at each respective time point using an approach described previously.[1] However, it was not possible to use the axial length to correct the transverse magnification of the B-scans collected during accommodation, given the need to adjust the axial length measurements to account for the influence of the changes in crystalline lens thickness.[2] Instead, the change in the magnification of the retinal en-face images obtained during accommodation compared to the images obtained during no accommodation was examined empirically by comparing the size of vascular landmarks in the retina using a custom written program (assuming that the retinal vasculature was not altered substantially with accommodation). This empirical approach showed that the retinal en-face images obtained during 5 D accommodation demand were on average 0.05 ± 0.26 % (range -0.49 to 0.89 %) magnified compared to the images obtained during no accommodation. These individual magnification differences were used to adjust the transverse magnification of the B-scans obtained during accommodation relative to those obtained during relaxed accommodation.

References:

1. Vincent SJ, Collins MJ, Read SA, et al. Retinal and choroidal thickness in myopic anisometropia. Invest Ophthalmol Vis Sci. 2013; 54: 2445-56.

2. Atchison DA, Smith G. Possible errors in determining axial length changes during accommodation with the IOLMaster. Optom Vis Sci. 2004; 81: 283-286.
